# Analysis of Fall Events, Physical Fitness, and Gait Speed According to Fall Risk in Older Korean Women

**DOI:** 10.3390/healthcare10101936

**Published:** 2022-10-02

**Authors:** Byung-Kun Lee, Wi-Young So, Hyun-Joo Kang

**Affiliations:** 1 Department of Lifetime Sports & Leisure, Sangmyung University, Cheonan 31066, Korea; 2 Sports Medicine Major, College of Humanities and Arts, Korea National University of Transportation, Chungju-si 27469, Korea; 3 Department of Sports Medicine, Soonchunhyang University, Asan-si 31538, Korea

**Keywords:** agility, aging, Berg Balance Scale, falls, flat ground, gait speed, mobility, older adults, physical fitness

## Abstract

The present study aimed to investigate the associations between fall risk and previous fall events, physical fitness, and gait speeds on flat ground and during obstacle avoidance in older adult women in Korea. Data were analyzed for 148 women over 65 years of age, divided into low (n = 52) and high Berg Balance Scale (BBS) score groups (n = 96). Physical fitness was assessed using arm curl, chair stand, 2 min step, chair sit-and-reach, timed up-and-go, and single-leg stance tests. Gait speed was measured on flat ground and during obstacle avoidance (5 cm, 10% of height, 30 cm). The incidence of falls was 18.2% lower among participants with BBS scores above the mean than in those with scores below the mean (*p* < 0.05). Furthermore, the two groups exhibited significant differences in the chair stand, chair sit-and-reach, timed up-and-go, and single-leg stance test results. The upper BBS group appeared faster at all four gait speeds, and participants in the upper BBS group were less likely to exhibit lower physical fitness in each test, with odds ratios (ORs) ranging from 0.227 to 0.447. The upper BBS group was also less likely to exhibit lower gait speed in most conditions, with ORs ranging from 0.327 to 0.516. Further studies should consider exercise programs that promote balance, muscular strength, and proprioception to lower the risk of falling in older adults.

## 1. Introduction

As of 2020, the average life expectancy at birth in South Korea was 83.5 years, which is the second highest among all Organization for Economic Co-operation and Development countries [1]. Continued increases in life expectancy are likely, given the improvements in living environments and advancements in medical technology. However, the disability-free life expectancy, which excludes periods of inactivity owing to disease or injury, is only 66.3 years among South Korean citizens [1]. The rapid aging of the population and this gap of approximately 17 years may contribute to exponential increases in medical expenses, resulting in a substantial economic burden [2].

Age-related musculoskeletal physical changes can increase the risk of physical impairment and may lead to poor posture in older adults. Among these changes, decreased range of motion in the knee and hip joints can lead to stiffened movements and altered gait patterns, thereby contributing to balance impairments. Such impairments in balance increase the risk of falling, which is associated with various adverse outcomes, including increased mortality [3]. In addition to limitations on physical activity, falls contribute to the maintenance of a vicious cycle of progressive instability, further increasing the risk of fall recurrence [3]. As such, the risk of falling is an important consideration in the management of older adult patients.

Physical fitness parameters such as balance, mobility, and gait pattern are known predictors of fall risk. The Berg Balance Scale (BBS) is an efficient, low-cost, and easily interpreted tool for the functional assessment of fall risk and daily living activities impairment [4]. As such, numerous studies have utilized the BBS to examine the relationships between fall risk and patient outcomes [5,6,7].

Many instruments have been developed to assess balance and predict falls in older adults. One of the most reliable and valid outcome measures developed today that has been tested in a variety of settings with different populations and diagnoses is BBS [8]. The BBS has been well established as a valid measure for predicting falls in a variety of patient populations. It has been validated as a predictor of length of hospital stay and discharge destination in stroke rehabilitation [9,10] and as a predictor of length of hospital stay and outcomes in acute inpatient rehabilitation when used in conjunction with the functional independence measure [11]. According to the Park SH study that assessed which tools best predict the risk of falls in the older adults, the BBS showed both pooled sensitivity and a pooled specificity of >0.7. Thus, the BBS was found to be the most useful tool for identifying the older adults with low fall risk [12]. Given that physical fitness facilitates regular physical activity, adequate functioning in activities of daily living, and healthy aging, such assessments are particularly relevant for older adults.

Age-related changes in cardiorespiratory fitness lead to the structural and functional deterioration of the cardiovascular and respiratory systems, negatively affecting the interactions among various organs and tissues while contributing to physical disability and cognitive impairment [13,14]. Progressive decreases in skeletal muscle mass, strength, and physical function caused by aging may lead to sarcopenia, loss of independence, and balance and mobility issues. Furthermore, age-related changes in the collagen structure of connective tissue, increased stiffness in the joints, loss of tissue elasticity, and thickening of the joint tissues significantly reduce the range of motion, thereby leading to decreased flexibility [15]. These physical fitness deficits also increase the risk of falls, highlighting the need for a thorough investigation of the precise physical fitness parameters associated with fall risk.

In addition to physical fitness parameters, gait patterns, including gait speed under various environmental conditions, may also act as fall risk predictors. Yu et al. [16] reported that dorsiflexion of the foot when crossing an obstacle could reduce fall risk. An 8-year prospective cohort study investigating whether changes in cognitive performance can predict falls noted that cognitive performance was associated with falling in adults aged >65 years; thus, cognitive performance should be assessed in clinical practice when evaluating fall risk [17]. In previous studies, various predictors were suggested as potential indicators of fall risk, such as female sex, past falling event, balance impairment, muscle weakness, visual impairment, gait disturbance, depression, and dizziness [18]. Despite an increased focus on factors that may predict fall risk and aid fall prevention, the evidence to date is insufficient. Therefore, to provide further evidence-based data for the development of safe and effective exercise programs for reducing fall risk, the present study aimed to investigate the associations between fall risk and previous fall events, physical fitness, and gait speeds on flat ground and during obstacle avoidance in older adult women in Korea. In addition, we aimed to analyze the odds ratios for upper and lower physical fitness associated with fall risk in different gait speed groups.

## 2. Materials and Methods

### 2.1. Study Design and Procedure

This study was conducted in accordance with the Declaration of Helsinki and was approved by the Ethics Committee of Soonchunhyang University (No. 1040875-202103-SB-022).

### 2.2. Participants

Women aged 65 years or older with the ability to engage in independent physical activities were recruited via notices posted at the Asan Senior Welfare Center in Asan-si, Korea. Patients with a history of heart surgery, myocardial infarction, stroke diagnosis and treatment within the last 6 months, serious orthopedic problems such as current fractures or deformities, severe osteoarthritis, severe visual impairment, visual field loss, and uncontrolled hypertension were excluded. After a sufficient explanation of the purpose and necessity of the study, 148 participants who voluntarily provided consent were included. The participants were divided into two groups on the basis of the average BBS score. The lower BBS group comprised participants with a score below 52 out of 56 points, and the upper BBS group comprised participants with a score above 53 out of 56 points. This result was very similar to the cutoff score of previous studies [19,20,21].

In an assessment of which cutoff scores on the BBS best predicted the risk of falling, Muir et al. [19] reported an area under the receiver operating characteristic (ROC) curve of 0.68 for multiple falls with a cutoff score of ≤53. As determined from the ROC analysis, the optimal single cutoff value for multiple falls was 53. The authors recommended a higher cutoff score of 53 to identify participants with a history of multiple falls and 54 to identify any older adult at risk of falls for optimal fall prediction sensitivity [19]. Wang et al. [20] performed an item analysis of the BBS with 268 community-dwelling older adults Taiwanese residents (age 73.84 ± 5.2). The mean (standard deviation) score was 53.3 (4.1). The item response profile showed that more than 90% of the participants attained the highest score on all [20]. According to Ersoy et al. [21], women aged ≥50 years who had a BBS score of ≤52 points (OR = 4.77, per additional point, 95% CI = 1.15–19.82, *p* = 0.031) performed best in the prediction of future falls.

### 2.3. BBS and Fall Event Test

The participants completed the 14-item Korean version of the Berg Balance Scale (K-BBS-14) [22] as part of the fall risk model developed by Lajoie and Gallagher [23]. This scale consists of the following 14 items related to balance-specific activities, ranging from sit-to-stand to standing on one leg: (1) sitting unsupported, (2) standing unsupported, (3) standing with eyes closed, (4) standing with feet together, (5) standing on one foot, (6) turning to look behind, (7) retrieving an object from the floor, (8) tandem standing, (9) reaching forward with an outstretched arm, (10) sitting to standing, (11) standing to sitting, (12) transfer, (13) turning 360°, and (14) stool stepping. All these items must be completed safely. Each item on the BBS-14 is rated from 0 (unable to try or need assistance to prevent falling) to 4 (able to lift the leg independently and hold for >10 s), resulting in a maximum possible score of 56. The BBS has been well established as a valid measure for predicting falls in a variety of patient populations. Moreover, moderate-to-high reliability has been established for BBS within a variety of settings and diagnoses. Relative intrarater reliability for the BBS was high (Interclass Correlation Coefficient [ICC] = 0.97) when used with participants in residential care facilities [24], and good interrater reliability (ICC = 0.87) was seen for the community-dwelling older adult Taiwanese residents [20]. Interrater and intrarater reliability were deemed excellent in acute stroke and older adult populations in a study by Berg et al. [25]. One of the most reliable and valid outcome measures tested in a variety of settings with different populations and diagnoses developed today is the BBS [8]. A fall-related questionnaire was used to investigate the fall, and the range of falls was defined as slip, tripping, and a bump.

### 2.4. Body Composition

Bioelectrical impedance analysis using the InBody 270 multifrequency system (Biospace, Seoul, Korea) was conducted to measure body composition on the basis of body fat, muscle mass, and body mass index.

### 2.5. Physical Fitness Test

Participants underwent a senior physical fitness test [26] and an assessment of physical ability (i.e., functional outcomes). The physical fitness test protocol consisted of six phases. Before the test, the physical conditions necessary for the application of the physical fitness test protocol were established. The test was completed within 30–40 min. Each participant performed the test after a warm-up exercise. Phase 1 consisted of an arm curl test for the assessment of upper body strength; a participant was instructed to perform as many forearm curls as possible with 2-kg dumbbells in 30 s. Phase 2 consisted of a chair stand test, which was used to evaluate lower body strength. In this test, participants were instructed to stand from a chair and sit back down repeatedly for 30 s, and the total number of stands was recorded. Phase 3 consisted of a 2 min step test to evaluate cardiorespiratory fitness. Participants were instructed to walk along a straight line, and the number of full steps completed within 2 min was recorded. Phase 4 consisted of the timed up-and-go test to evaluate agility and dynamic balance. For this test, participants were instructed to rise from a chair of usual height and with a backrest, walk over 3 m at their usual gait speed, return to the chair, and sit back on it. Phase 5 consisted of a chair sit-and-reach test, which was used to assess lower-body flexibility. While seated on the edge of a chair, participants were instructed to keep one foot flat on the floor while extending the other leg forward with the knee straight. One hand was placed on top of the other with the tips of the middle fingers aligned. During exhalation, participants reached forward toward the toes, hinging at the hip. The distance between the fingertips and toes was then measured. Phase 6 consisted of a single-leg stance test conducted with the eyes open. The time spent in a stable standing position on the dominant foot while maintaining a vertical thigh position parallel to the standing leg was measured for each participant. There was no time limit, and it measured the ability to do it to the maximum. The period of single-leg stance was 44.4 ± 2.4 s, and it lasted up to 115 s. The better of two measurements was taken as the value for analysis in each participant.

### 2.6. Gait Parameter Measurements

A quantitative gait speed assessment was performed using a 9 × 1 m walkway embedded with pressure sensors (IB-GAIT, Inbody, Seoul, Korea). Participants were instructed to walk along the walkway at their usual pace. They were asked to take some strides before striking the ground and continue walking after reaching the end of the walkway to avoid the influence of deceleration. Before the actual measurement, all participants were allowed to participate in a practice trial. Obstacle gait was measured while crossing obstacles of 5 cm, 10% of the participant’s height (10% HT), and 30 cm. Obstacle gait speed was calculated from the time when the heel of the stepping foot touched the ground before crossing the obstacle (heel strike) to the point when the raised foot crossed the obstacle.

### 2.7. Statistical Analysis

SPSS version 23.0 for Windows (IBM Corp., Armonk, NY, USA) was used for statistical analyses, with a statistical significance level of α = 0.05. Chi-square analyses were used to examine the differences in fall events between the upper and lower BBS groups. Independent *t*-tests were used to compare physical fitness, flat ground gait speed, and obstacle gait speed in the upper and lower BBS groups. Logistic regression analysis was performed to compare the relative risk based on physical fitness and obstacle gait speed between the lower and upper BBS groups.

## 3. Results

### 3.1. Physical Characteristics of the Study Participants

The physical characteristics of the participants are presented in Table 1. The upper and lower BBS groups showed significant differences only in age (*p* < 0.001).

### 3.2. Comparison of Fall Events According to Fall Risk Level (BBS)

The percentages of participants experiencing falls in the lower and upper BBS groups were 59.1% and 18.2%, respectively (total: 40.9%). Fall events were not reported in 31.0% and 69.0% of the lower and upper BBS groups, respectively. Chi-square analysis revealed a significant difference in fall events between the upper and lower BBS groups (*p* = 0.015) (Table 2).

### 3.3. Comparison of Physical Fitness and Gait Speed in Upper and Lower Fall Risk Groups

Significant differences between the upper and lower BBS groups were observed in the chair stand (*p* = 0.001), chair sit-and-reach (*p* = 0.013), timed up-and-go (*p* < 0.001), and single-leg stance with eyes open tests (*p* < 0.001). These results indicate that patients with high and low fall risk exhibited significant differences in lower extremity strength, flexibility, and balance (Table 3). However, no significant differences in the arm curl test (an index of muscular strength in the upper extremities) or the 2 min step test (an index of cardiorespiratory endurance) were observed between the groups.

The upper and lower BBS groups exhibited significant differences in flat ground gait speed (*p* = 0.019), 5 cm obstacle gait speed (*p* = 0.005), 10% HT obstacle gait speed (*p* = 0.036), and 30 cm obstacle gait speed (*p* = 0.010). Gait speed gradually decreased for the flat ground, 5 cm obstacle, 10%HT, and 30 cm obstacle conditions. The upper BBS group was faster than the lower BBS group by 0.225 m/s in the flat ground condition, 0.119 m/s in the 5 cm obstacle condition, 0.089 m/s in the 10% HT obstacle condition, and 0.098 m/s in the 30 cm obstacle condition (Table 4).

### 3.4. Relative Risk for Physical Fitness and Gait Speed According to Fall RISK Level (BBS)

Logistic regression analysis was performed to determine thresholds for physical fitness according to fall risk (BBS). The upper BBS group was less likely to be included in the lower physical fitness group, and the odds ratios were 0.368 in the chair stand test (95% confidence interval [CI]: 0.177–0.766, *p* = 0.007), 0.447 in chair sit-and-reach test (95% CI: 0.221–0.904, *p* = 0.025), 0.323 in the timed up-and-go test (95% CI: 0.159–0.652, *p* = 0.002), and 0.227 in the single-leg stance with eyes open test (95% CI: 0.104–0.494, *p* < 0.001), with significant differences in all comparisons (Table 5). However, the odds ratios for the arm curl test (1.102; 95% CI: 0.559–2.171, *p* = 0.779) and the 2 min step test (0.870; 95% CI: 0.429–1.762, *p* = 0.699) were not significant. When the number of chair stands was at least 14.58 each/30 s, the lower BBS odds ratio decreased to 0.368. Further, when the chair sit-and-reach distance was at least 12.49 cm, the lower BBS odds ratio decreased to 0.447. When the timed up-and-go result was 7.59 s or less, the lower BBS odds ratio decreased to 0.323. When the duration in the single-leg stance with eyes opened test was 28.99 s or more, the lower BBS odds ratio decreased to 0.227. There were no significant differences in the odds ratios for other physical fitness indices, which ranged from 0.870 to 1.102 (Table 5).

Logistic regression analysis was performed to determine the relative risk for different gait speed levels on flat ground and during obstacle avoidance, according to the fall risk level based on the BBS score. The upper BBS group was less likely to be included in the lower gait speed group, with ORs of 0.327 for gait speed on flat ground (95% CI: 0.167–0.640, *p* = 0.001), 0.605 for gait speed during the 5 cm obstacle condition (95% CI: 0.335–1.092, *p* = 0.095), 0.516 for gait speed in the 10% HT obstacle condition (95% CI: 0.283–0.940, *p* = 0.031), and 0.462 for gait speed in the 30 cm obstacle condition (95% CI: 0.251–0.850, *p* = 0.013). All differences were significant except for the 5 cm obstacle condition (Table 6). When the gait speed on flat ground was 1.28 m/s or more, the odds ratio in the lower BBS group decreased to 0.327. Further, when the gait speed on flat ground was 1.18 m/s or more, the odds ratio in the lower BBS group decreased to 0.605. When gait speed in the 10% HT obstacle condition was 1.02 m/s or more, the odds ratio in the lower BBS decreased to 0.516. When gait speed in the 30 cm obstacle gait speed was 0.82 m/s or more, the odds ratio in the lower BBS group decreased to 0.462 (Table 6).

## 4. Discussion

Agility and dynamic balance issues are the main causes of falls among older adults. Rikli and Jones [26] reported that agility and dynamic equilibrium are related to gait speed, in turn affecting activities of daily living. Decreases in gait speed and mobility have also been reported as risk factors associated with falls [5]. Approximately 50% of fall accidents have been reported to occur during tasks that require static and dynamic balance, such as the start and end of gait and rotation in front of a wall [27]. Neuls et al. [8] said that it was recommended to check the score of a subject who needs to use a mobile device because of an increased risk of falling.

In this study, the average age of the participants in the lower BBS group was 74.2 ± 5.8 years, that of the participants in the upper BBS group was 71.2 ± 4.9 years, and the participants in the upper BBS group were relatively younger than those in the lower BBS group (Table 1). Over one-third of adults aged 65 or older and half of those aged 80 years or older commonly experience falls [3,28]. The frequency of falls increases with age, and women are at greater risk than men [29]. De Rekeneire et al. [30] reported that, among adults aged 70 to 80, 24.1% of women and 18.3% of men experienced a fall within the past year. Liang et al. [31] reported that 60 (26.1%) of 230 men aged 80 years and older in Taiwan had experienced a fall, while Bath et al. reported a fall rate of 26.4% [32]. Ribom et al. [33] reported that, among 3014 Swedish men aged 69–80 years, 16.5% had at least one fall in the previous year, while 7.4% had at least two. The authors further reported that those who had experienced a fall had lower scores for all physical fitness parameters than those who had not. In the current study of older women in Korea, the fall rate was 14.9%, which is slightly lower than the rates reported by Rekeneire et al. [30], Liang et al. [31], and Bath et al. [32] but similar to that reported by Ribom et al. [33]. These discrepancies may be due to differences in study designs. The relatively low rate reported by Ribom et al. [33] may be related to the need to obtain measurements over several hours; one-year recall studies may better reflect actual falls than long-term recall studies. Differences in the ages of the included participants may also have contributed to these discrepancies.

Muscle weakness, which leads to decreased physical function and mobility, can lead to falls and fall-related injuries, which can, in turn, increase the risk of mortality. Previous studies have emphasized the importance of fall-related physical fitness in preventing falls [18]. Lower body muscular fitness is an essential component of physical functioning when standing up from a chair, going up and down stairs, shopping, and traveling in daily life [34]. Studies have reported that chair-stand test performance is associated with normal age-related functional decline [35] and fall risk [36] and can predict fallers and nonfallers [37]. In this study, lower body muscle strength determined based on the chair stand and timed up-and-go tests was significantly lower in the lower BBS group than in the higher BBS group (Table 3). Given the rapid aging of the general population and increases in fall incidence, these findings highlight the need for interventions that promote regular physical activity and lower body strength to improve functioning in daily activities and lower the risk of falls among older adults [38].

Flexibility is an indicator of the ability to perform various activities, such as maintaining correct posture, changing clothes, and walking with a normal gait. In this study, the lower BBS group had significantly lower scores in the chair sit-and-reach test, which measures lower body flexibility (Table 3). Flexibility refers to the maximal joint range of motion that body tissues can intrinsically achieve without injury [39]. With age, changes in the musculoskeletal system reduce the joints’ range of motion, resulting in reduced mobility and stride length, which may increase the risk of falls during gait activities. Medical conditions associated with dizziness, such as hypertension, hypotension, or diabetes, have been associated with an increased risk of falls, and this risk can be nine times higher than that in the general older adult population with joint problems or arthritis [40]. Therefore, activities that improve flexibility, such as stretching exercises and yoga, should be included when designing interventions to mitigate fall risk in older adults.

Degeneration of the musculoskeletal system adversely affects not only the range of motion in the joints but also balance, owing to progressive decreases in physical ability [41]. In this study, we utilize the timed up-and-go test to assess the speed, agility, and dynamic balance in older adult women. Agility and dynamic balance are essential for safe, smooth movements in daily life, such as getting off the bus, going to the bathroom, answering a phone call, and opening the door. Research has indicated that the fall risk increases as the time required for the timed up-and-go test increases [42]. As in previous studies, the times in the timed up-and-go test and single-leg stance test for static balance were significantly shorter in the upper BBS group than in the lower BBS group (Table 3).

A previous study conducted among community-dwelling older adults at high risk for falls reported that a 6-month therapeutically designed Tai Ji Quan balance training intervention was more effective in reducing the incidence of falls than stretching exercise or multicomponent exercise [43]. Balance can be defined as the ability to control posture. As such, Tai Chi exercises may aid in increasing dynamic balance and muscle strength in the lower body. Indeed, previous studies have reported that various Tai Chi movements can prevent falls in older adults by enhancing not only balance but also proprioception and gait ability [44]. Chan et al. further reported that men with greater leg and grip strength had a significantly lower risk of falling than those with a relatively lower strength (ORs for highest quartile vs. lowest quartile for relative risk: leg strength, 0.82; grip strength, 0.76) [45].

In a study that performed logistic regression analysis to confirm the main factors associated with fall risk, participants with times of less than 8.14 s in the 8-foot up-and-go test, an indicator of agility and balance, had ORs for maximum BBS scores that were 11 times higher than those in the reference group. Toraman et al. [46] reported an average BBS value as high as 54 out of 56 points because they excluded participants who had experienced a fall in an effort to target older adults with good physical functioning. Park et al. analyzed factors that can positively affect functional fitness and mood in independent older adults living in Korea [47]. The high-risk threshold for the chair stand test for women aged 70–75 was 11 each/30 s. This was lower than the overall average of 14.58 ± 5.04 each/30 s for women in the current study and lower than the average in the lower BBS group (12.75 ± 4.25 s). When the chair stand result is 11 each/30 s or less, independent living can be considered difficult. Further, when the result was 14.58 ± 5.04 each/30 s or more, the OR for the lower BBS group decreased to 0.368, suggesting a relationship between lower extremity muscular fitness and falls. Kang and Lee [3] reported that the OR for fall events in the low (<30%) vs. medium gait speed (>30%) group was 2.844 (*p* < 0.05) on flat ground, indicating an increased risk of falls in the low gait speed group. ORs for the 5 cm obstacle and 30 cm obstacle conditions were 3.585 (*p* < 0.05) and 4.877 (*p* < 0.01). Thus, as the height of the obstacle increased, the OR for falls in the low gait speed group tended to increase. Kang and Lee also reported that when the analyses for flat ground and obstacle conditions were adjusted for age and body mass index, fall risk rapidly increased as the height of the obstacle increased among individuals with low gait speed [3].

Ribom et al. [33] reported that 9.3% of fallers were in the low physical fitness group, defined based on a result ≤3 standard deviations below the mean in the timed chair-stand test, while only 0.3% of nonfallers were in the low physical fitness group (*p* < 0.001). The OR for falling in the low physical fitness group was 3.41 (*p* < 0.001). In the 20 cm narrow walk test, 13.1% of fallers were in the low physical fitness group versus only 0.1% of nonfallers (*p* < 0.001). The OR for falling among those with low physical strength was 2.46 (*p* < 0.001). In this study, the ORs for fall risk (BBS) among those with low physical fitness in each test (chair stand, chair sit-and-reach, timed up-and-go, and single-leg stance) ranged from 0.227 to 0.447, with those in the lower gait speed group ranging from 0.327 to 0.605. Thus, the current results support a meaningful relationship between BBS and these fitness indicators (Table 5 and Table 6). Nonetheless, further studies are required, given that falls may represent both a cause and consequence of altered physical fitness. Our results suggest that BBS scores above the mean can decrease the ORs for poor balance and flexibility from 0.227 to 0.605, suggesting that the BBS can be used to guide fall prevention strategies.

This study has several limitations, as we only included 148 women aged 65 years or older; thus, first, considering that there are approximately 8.12 million older adults in Korea, the sample size was relatively small. Second, this study was restricted to older adults visiting a senior welfare center located in Asan-si, which limits the generalizability of the results. Third, only the BBS was used to discriminate fall risk levels in the current study, which may have introduced bias. Finally, this study only included women; therefore, future research should include men in a well-designed study on this subject.

## 5. Conclusions

The current study revealed that the incidence of falls was 18.2% lower in those with BBS scores above the mean than in those with BBS scores below the mean, representing a significant difference. Furthermore, the two groups exhibited significant differences in the chair stand, chair sit-and-reach, timed up-and-go, and single-leg stance test results. The participants in the upper BBS group were faster at all four gait speeds and less likely to be included in the group with lower physical fitness in each test, with ORs ranging from 0.227 to 0.447. These participants were also less likely to be included in the group with lower gait speed for most conditions, with ORs ranging from 0.327 to 0.516. On the basis of these findings, rehabilitation programs should consider these fall-related physical fitness factors as well as gait speeds under various conditions when designing interventions for fall prevention. Since the risk of a fall increases predominantly when an obstacle appears or during walking, further studies should consider exercise programs that promote improvements in balance, muscular strength, and proprioception to lower the risk of falling.

## Figures and Tables

**Table 1:** Physical characteristics of the study participants.

Group	Age (Years)	Weight (kg)	Height (cm)	Body Mass Index (kg/m^2^)
Lower BBS (n = 52)	74.2 ± 5.8	59.0 ± 8.0	153.7 ± 5.1	24.9 ± 3.0
Upper BBS (n = 96)	71.2 ± 4.9	57.4 ± 7.8	154.3 ± 4.6	24.1 ± 3.0
Total (n = 148)	72.2 ± 5.2	58.0 ± 7.9	154.1 ± 4.8	24.3 ± 3.0
*t*	3.484	1.174	0.676	1.590
*p*	0.001 ***	0.243	0.500	0.114

BBS—Berg Balance Scale; Lower BBS shows values below mean values (52.3 points), Upper BBS shows values above mean values (52.3 pinots), BMI—body mass index, and *** is *p* < 0.001, tested via independent *t*-test.

**Table 2:** Comparison of fall events according to fall risk level (BBS).

Fall Event	Variables	Fall Risk Level		Total
		Lower BBS (n = 52)	Upper BBS (n = 96)	
Non-fallers	Frequency	39	87	126
	No fall event %	31.0%	69.0%	100.0%
	BBS group %	75.0%	90.6%	85.1%
	Total %	26.4%	58.8%	85.1%
Fallers	Frequency	13	9	22
	Fall event e %	59.1%	40.9%	100.0%
	BBS group %	25.0%	9.4%	14.9%
	Total %	8.8%	6.1%	14.9%
Total	Frequency	52	96	148
	Fall and non-fall event %	35.1%	64.9%	100.0%
	BBS group %	100.0%	100.0%	100.0%
	Total %	35.1%	64.9%	100.0%

BBS—Berg Balance Scale; Lower BBS shows values below mean (52.3 points), Upper BBS shows values above mean (52.3 points), χ2 = 6.500, and *p* = 0.015, tested via Pearson’s chi-square analysis.

**Table 3:** Comparison of physical fitness according to fall risk level (BBS).

Physical Fitness	Fall Risk Levels		Total	t	*p*
	Lower BBS(n = 52)	Upper BBS(n = 96)			
Arm curl (each/30 s)	16.54 ± 4.14	17.26 ± 4.62	17.01 ± 4.45	0.940	0.349
Chair stand (each/30 s)	12.75 ± 4.25	15.57 ± 5.17	14.58 ± 5.04	3.365	0.001 **
2 min step test (steps)	134.73 ± 54.41	131.39 ± 42.61	132.55 ± 46.88	0.380	0.705
Chair sit-and-reach (cm)	10.21 ± 7.18	13.73 ± 8.55	12.49 ± 8.25	2.523	0.013 *
Timed up-and-go (s)	8.46 ± 2.11	7.13 ± 2.04	7.59 ± 2.15	3.714	<0.001 ***
Single-leg stance with eyes open (s)	17.74 ± 20.65	35.08 ± 24.56	28.99 ± 24.63	4.556	<0.001 ***

BBS—Berg Balance Scale; Lower BBS shows values below mean (52.3 points), Upper BBS shows values above mean (52.3 points), * is *p* < 0.05, ** *p* < 0.01, and *** *p* < 0.001, tested via independent *t*-test.

**Table 4:** Comparison of flat ground and obstacle gait speeds, according to fall risk level (BBS).

Gait Speed	Fall Risk Level		Total	t	*p*
	Lower BBS (n = 52)	Upper BBS (n = 96)			
Flat ground gait speed (m/s)	1.14 ± 0.20	1.37 ± 0.66	1.28 ± 0.55	2.365	0.019 *
5 cm obstacle gait speed (m/s)	1.11 ± 0.24	1.23 ± 0.23	1.18 ± 0.23	2.863	0.005 **
10% HT obstacle gait speed (m/s)#	0.96 ± 0.25	1.05 ± 0.23	1.02 ± 0.23	2.115	0.036 *
30 cm obstacle gait speed (m/s)	0.76 ± 0.21	0.86 ± 0.21	0.82 ± 0.21	2.617	0.010 *

BBS—Berg Balance Scale; Lower BBS shows values below mean (52.3 points); Upper BBS shows values above mean (52.3 points); # is gait speed with an obstacle at 10% of the participant’s height; * is *p* < 0.05, ** is *p* < 0.01, tested via independent *t*-test.

**Table 5:** The relative risk for different levels of physical fitness, according to fall risk levels (BBS).

Variables	OR	95% CI	*p*
Arm curl (each/30 s)	1.102	0.559–2.171	0.779
Chair stand (each/30 s)	0.368	0.177–0.766	0.007 **
2 min step test (steps)	0.870	0.429–1.762	0.699
Chair sit-and-reach (cm)	0.447	0.221–0.904	0.025 *
Timed up-and-go (s)#	0.323	0.159–0.652	0.002 **
Single-leg stance with eyes open (s)	0.227	0.104–0.494	<0.001 ***

BBS—Berg Balance Scale; # is reverse cording of timed up-and-go (s); OR—odds ratio; CI—confidence interval; * is *p* < 0.05, ** *p* < 0.01, *** *p* < 0.001, tested via logistic regression analysis.

**Table 6:** The relative risk for different gait speeds in each condition, according to fall risk level (BBS).

Variables	OR	95% CI	*p*
Flat ground gait speed (m/s)	0.327	0.167–0.640	0.001 **
5 cm obstacle gait speed (m/s)	0.605	0.335–1.092	0.095
10% HT obstacle gait speed (m/s)#	0.516	0.283–0.940	0.031 *
30 cm obstacle gait speed (m/s)	0.462	0.251–0.850	0.013 *

BBS ± Berg Balance Scale; # is gait speed for an obstacle at 10% of the participant’s height; OR—odds ratio, CI—confidence interval; * is *p* < 0.05, ** *p* < 0.01, tested by logistic regression analysis.

## Data Availability

The data presented in this study are available upon request from the authors. Some variables were restricted to preserve the anonymity of study participants.

## References

[1] Korean Statistical Information Service (2020). Average Life Expectance at Birth in South Korea. Korean Statistical Information Service. http://kosis.kr/index/index.do.

[2] Korea Institute for Health and Social Affairs (2019). Social Problem-Solving Health and Medical Technology and Policy Tasks for Extending Health Lifetime.

[3] Kang H.J., Lee B.K. (2022). Comparison of gait variables of fallers and non-fallers and relative risk of falls according to gait speed during flat and obstacles gait in Korean elderly women. Exer. Sci..

[4] Berg K., Wood-Dauphinee S., Williams J.I., Cayton D. (1989). Measuring balance in the elderly: Preliminary development of an instrument. Phys. Ther. Can..

[5] Shumway-Cook A., Baldwin M., Polissar N.L., Gruber W. (1997). Predicting the probability for falls in community-dwelling older adults. Phys. Ther..

[6] Huang W.-N.W., Perera S., VanSwearingen J., Studenski S. (2010). Performance measures predict onset of activity of daily living difficulty in community-dwelling older adults. J. Am. Geriatr. Soc..

[7] Park S.-H., Lee Y.-S. (2017). The diagnostic accuracy of the Berg balance scale in predicting falls. West. J. Nurs. Res..

[8] Neuls P.D., Clark T.L., Van Heuklon N.C., Proctor J.E., Kilker B.J., Bieber M.E., Donlan A.V., Carr-Jules S.A., Neidel W.H., Newton R.A. (2011). Usefulness of the Berg Balance Scale to predict falls in the elderly. J. Geriatr. Phys. Ther..

[9] Wee J.Y., Bagg S.D., Palepu A. (1999). The Berg balance scale as a predictor of length of stay and discharge destination in an acute stroke rehabilitation setting. Arch. Phys. Med. Rehabil..

[10] Wee J.Y., Wong H., Palepu A. (2003). Validation of the Berg balance scale as a predictor of length of stay and discharge destination in stroke rehabilitation. Arch. Phys. Med. Rehabil..

[11] Juneja G., Czyrny J.J., Linn R.T. (1998). Admission balance and outcomes of patients admitted for acute inpatient rehabilitation. Am J. Phys. Med. Rehabil..

[12] Park S.H. (2018). Tools for assessing fall risk in the elderly: A systematic review and meta-analysis. Aging Clin. Exp. Res..

[13] American College of Sports Medicine (2018). ACSM’s Guidelines for Exercise Testing and Prescription.

[14] Eskurza I., Donato A.J., Moreau K.L., Seals D.R., Tanaka H. (2002). Changes in maximal aerobic capacity with age in endurance-trained women: 7yr follow up. J. Appl. Physiol..

[15] Korea Association of Geriatric Healthy Exercise (2015). Guidelines of Exercise Medicine for Older Adults.

[16] Yu Y.J., Lee K.K., Kim S.B. (2014). Effects of fear of falling and cognitive task on the obstacle gait in older adults. J. Korean Acad. Kinesiol..

[17] Anstey K.J., von Sanden C., Luszcz M.A. (2006). An 8-year prospective study of the relationship between cognitive performance and falling in very old adults. J. Am. Geriatr. Soc..

[18] Tareef A.-A. (2011). Falls in the elderly. Can. Fam. Physician.

[19] Muir S.W., Berg K., Chesworth B., Speechley M. (2008). Use of the Berg Balance Scale for Predicting Multiple Falls in Community-Dwelling Elderly People: A Prospective Study. Phys. Ther..

[20] Wang C.Y., Hsieh C.L., Olson S.L., Wang C.H., Sheu C.F., Liang C.C. (2006). Psychometric properties of the Berg balance scale in a community-dwelling elderly resident population in Taiwan. J. Formos. Med. Assoc..

[21] Ersoy Y., MacWalter R.S., Durmus B., Altay Z.E., Baysal O. (2009). Predictive Effects of Different Clinical Balance Measures and the Fear of Falling on Falls in Postmenopausal Women Aged 50 Years and Over. Gerontology.

[22] Jung H.Y., Park J.H., Shim J.J., Kim M.J., Hwang M.R., Kim S.H. (2006). Reliability test of Korean version of Berg balance scale. J. Korean Acad. Rehab. Med..

[23] Lajoie Y., Gallagher S.P. (2004). Predicting falls within the elderly community: Comparison of postural sway, reaction time, the Berg balance scale and the Activities-specific Balance Confidence (ABC) scale for comparing fallers and non-fallers. Arch. Gerontol. Geriatr..

[24] Conradsson M., Lundun-Olsson L., Lindelof N., Littbrand H., Malmqvist L., Gustafson Y., Rosendahl E. (2007). Berg balance scale: Intrarater test-retest reliability among older people dependent in activities of daily living and living in residential care facilities. Phys. Ther..

[25] Berg K.O., Williams J.I., Wood-Dauphinee S.L. (1995). The balance scale: Reliability assessment with elderly residents and patients with an acute stroke. Scand. J. Rehabi. Med..

[26] Rikli R.E., Jones C.J. (2001). Senior Fitness Test Manual.

[27] Duncan P.W., Studenski S., Chandler J., Prescott B. (1992). Functional reach: Predictive validity in a sample of elderly male veterans. J. Gerontol..

[28] Song S.-H. (2011). Classification, risk factor and assessment of fall. Geriatr. Rehabil..

[29] Kim S. (2012). Validity and Reliability of the Korean Version of the Activities Specific Balance Confidence Scale in Individuals with Stroke. Doctoral Thesis.

[30] De Rekeneire N., Visser M., Peila R., Nevitt M.C., Cauley J.A., Tylavsky F.A., Simonsick E.M., Harris T.B. (2003). Is a fall just a fall: Correlates of falling in healthy older persons. The Health, Aging, and Body Composition Study. J. Am. Geriatr. Soc..

[31] Liang C.K., Chou M.Y., Peng L.N., Liao M.C., Chu C.L., Lin Y.T., Chen L.K. (2014). Gait speed and risk assessment for falls among men aged 80 years and older: A prospective cohort study in Taiwan. Eur. Geriatr. Med..

[32] Bath P.A., Morgan K. (1999). Differential risk factors profiles indoor and outdoor falls in older people living at home in Nottingham, UK. Eur. J. Epidemiol..

[33] Ribom E.L., Grundberg E., Mallmin H., Ohlsson C., Lorenzon M., Orwoll E., Holmberg A.H., Mellstrom D., Ljunggren O., Karlsson M.K. (2008). Estimation of physical performance and measurements of habitual physical activity may capture men with high risk to fall--data from the Mr Os Sweden cohort. Arch. Gerontol. Geriatr..

[34] Choi H.J., Kim S.H., Kim S.Y., Lee B.K., Kang H.J. (2013). Comparison of body composition, functional fitness and foot pressure balance between muscle-strengthening type in older persons. A J. Kinesiol..

[35] Csuka M., McCarty D.J., Bohannon R.W. (1985). Simple method for measurement of lower extremity muscle strength. Am. J. Med..

[36] Alexander N.B., Schultz A.B., Warwick D.N. (1991). Rising from a chair: Effects of age and functional ability on performance biomechanics. J. Gerontoloty.

[37] MacRae P.G., Lacourse M., Moldavon R. (1992). Physical performance measures that predict faller status in community-dwelling older adults. J. Orthop. Sports Phy. Ther..

[38] Kang H.J., Kim S.B., Lee B.K. (2013). Effects of rhythmic exercise and strengthening-aerobic exercise on physical fitness and MVAS in older females. Asian J. Kinesiol..

[39] Nuzzo L.J. (2020). The case for retiring flexibility as a major component of physical fitness. Sports Med..

[40] Huang H.C., Gau M.I., Lin W.-C., George K. (2003). Assessing risk of falling in the adult. Public Health Nurs..

[41] Park J., Lee K.H. (2020). The effect of musculoskeletal disorders on body regions and pain levels in elderly people on dynamic balance ability. J. Mens Health.

[42] Cebolla E.C., Rodacki A.L.F., Bento P.C.B. (2015). Balance, gait, functionality and strength: Comparison between elderly fallers and non-fallers. Braz. J. Phys. Ther..

[43] Li F., Harmer P., Fitzgerald K., Eckstrom E., Akers L., Chou L.-S., Pidgeon D., Voit J., Winters-Stone K. (2018). Effectiveness of a therapeutic tai ji quan intervention vs a multimodal exercise intervention to prevent falls among older adults at high risk of falling: A randomized clinical trial. JAMA Intern. Med..

[44] Zeeuwe P.E.M., Verhagen A., Bierma-Zeinstra S., van Rossum E., Faber M.J., Koes B.W. (2006). The effect of tai chi chuan in reducing falls among elderly people: Design of a randomized clinical trial in the Netherlands [ISRCTN98840266]. BMC Geriatr..

[45] Chan B.K.S., Marshall L.M., Winters K.M., Faulkner K.A., Schwartz A.V. (2007). Incident fall risk, and physical activity and physical performance among older men: The Osteoporotic Fractures in Men Study. Am. J. Epidemiol..

[46] Toraman A., Yıldırım N.U. (2010). The falling risk and physical fitness in older people. Arch. Gerontol. Geriatr..

[47] Park S., Ko B.-G., Song H.-S., Song J.-H., Lee M., Jae S.Y., Jeon J., Kwon J.P.S., Lee J., Park S.H. (2018). Development of criterion referenced health fitness standards for chronic disease prevention in Korean adults: The Korea Institute of Sport Science Fitness Standards Study (KISS FitS). Kor. J. Sport Stud..

